# Oral manifestations of COVID‐19 patients: An online survey of the Egyptian population

**DOI:** 10.1002/cre2.429

**Published:** 2021-05-01

**Authors:** Dina M. El Kady, Esraa Ahmad Gomaa, Walid Shaban Abdella, Reham Ashraf Hussien, Rawda H. Abd ElAziz, Ahmad G. A. Khater

**Affiliations:** ^1^ Conservative Dentistry Department Cairo University Egypt; ^2^ Oral Radiology Department Egyptian Russian University Egypt; ^3^ Faculty of Medicine Al‐Azhar University Damietta Egypt; ^4^ Oral and Maxillofacial Radiology Department Cairo University Egypt; ^5^ Faculty of Oral and Dental Medicine Ahram Canadian University Egypt

**Keywords:** coronavirus disease 2019, COVID‐19, oral health, oral lesions, SARS‐CoV‐2

## Abstract

**Objectives:**

This pilot survey aims to study the oral manifestations associated with COVID‐19 infection and report the prevalence of oral signs and symptoms in COVID‐19 patients.

**Materials and Methods:**

From May 15 to June 10, 2020, we used an online questionnaire containing the oral manifestations that are expected to be associated with the COVID‐19 infection. Adults in our survey who have been diagnosed with COVID‐19 positive were confirmed with reverse transcriptase PCR (RT‐PCR), and isolated in various hospitals in Cairo, Egypt.

**Results:**

This pilot study included 58 (53.4% males and 46.6% females) COVID‐19 patients ages 18–46 years, and 13 (22.4%) were healthcare workers. Our results showed that 67.2% of the patients had at least one manifestation related to the oral cavity and salivary glands, and 32.8% (*n* = 19) did not have any symptoms associated with the oral cavity. The highest prevalence symptoms were dry mouth 39.7% (*n* = 23), gustatory dysfunction as 34.5% (n = 20) loss of salt sensation, 29.3% (*n* = 17) loss of sweet sensation, and 25.9% (*n* = 15) altered food taste, while the least prevalent symptoms were tongue redness 8.8% (*n* = 5), and gingival bleeding 7% (*n* = 4). The most frequently associated symptoms were loss of salt and sweetness, as reported by 27.6% of the participants. However, there was no significant association between the incidence of oral symptoms and demographic data (age, gender, or job) of the patients (*p* > 0.05).

**Conclusions:**

Based on limited data, COVID‐19 significantly impacts the oral cavity and salivary glands, as salivary gland‐related symptoms and taste disorders are highly prevalent in COVID‐19 patients.

## 1 INTRODUCTION

The novel coronavirus disease 2019 (COVID‐19) is a human‐to‐human transmitted disease caused by one of the coronaviruses, which are a large family of viruses that can cause serious disorders such as Severe Acute Respiratory Syndrome (SARS) and Middle East Respiratory Syndrome (MERS; Dar Odeh et al., 2020; Fini, 2020; Speth et al., 2020). The World Health Organization (WHO) recognized COVID‐19 as a pandemic disease, with most countries reporting significant numbers of infected people and deaths as of December 2019 (Organization, 2020).

The typical clinical symptoms of COVID‐19 patients were fever, cough, shortness of breath, and myalgia or weakness with abnormal chest CT, while the less prevalent symptoms were sputum formation, headache, hemoptysis, and diarrhea. Often, alteration or loss of taste and smell sensation has been observed (Guan et al., 2020; Huang et al., 2020; Lechien et al., 2020; Vinayachandran & Balasubramanian, 2020).

Coronavirus attacks human cells by angiotensin‐converting enzyme 2 (ACE2) receptors since the current evidence indicated that ACE2 acts as the primary host cell receptor for severe acute respiratory syndrome coronavirus 2 (SARS‐CoV‐2; Zou et al., 2020). As such, the virus will bind to ACE2 using the spike‐like protein on its surface, and ACE2 will serve as a cellular portal for viral entry into the cell to cause COVID‐19 infection (Ciaglia et al., 2020). Hence, organs with high ACE2 expression (e.g., lung) can become target cells during SARS‐CoV‐2 infection that cause inflammatory reactions in related organs and tissues, such as salivary glands and tongue, which could explain the occurrence of both loss of taste and oral ulceration due to destruction of keratinocytes and oral fibroblasts (Zhou et al., 2020). Otherwise, high viral load in the saliva and nasal secretion can be a pathogenic factor involved in developing the oral changes associated with COVID‐19 infection, which indicates the direct effect of the virus on the oral tissues (Cruz Tapia et al., 2020).

On the other side, these effects may be attributed to the virus's indirect influence on the immune system, contributing to other opportunistic infections, such as recurrent herpes simplex virus (HSV‐1) infection and oral ulcerations (Brandão et al., 2020; Dziedzic & Wojtyczka, 2020).

COVID‐19 has two routes of transmission, either directly or indirectly. It can be transmitted indirectly through saliva, while it may be spread directly by coughing, sneezing, and droplet inhalation or via direct contact with oral, nasal, and ocular mucous membranes (Peng et al., 2020; Speth et al., 2020).

The oral cavity is known to be an indicative mirror reflecting the underlying health condition. Therefore, careful examination of the mouth will assist in early diagnosis and treatment since some oral symptoms (e.g., oral ulcerations, gingival bleeding, dry mouth, and oral discomfort, halitosis, burning sensation, or difficulty swallowing) that can be associated with certain systemic disorders (Chi et al., 2010). Several studies have considered oral transmission to be one of the main routes of COVID‐19 infection (Gu et al., 2020; Hindson, 2020; Yeo et al., 2020). Even though many studies addressed the validity of saliva in the COVID‐19 diagnosis (Fernandes et al., 2020; Pedrosa et al., 2020; Sabino‐Silva et al., 2020; To et al., 2020), as well as the precautions and implications for dental practice during the COVID‐19 pandemic era (Coulthard, 2020; Ge et al., 2020; Sabino‐Silva et al., 2020; Spagnuolo et al., 2020), there is still a gap of knowledge regarding the oral manifestations related to COVID‐19 and its impact on the oral cavity. Therefore, this pilot survey aims to study the oral manifestations associated with COVID‐19 infection and report the superiority of oral signs and symptoms in COVID‐19 patients.

## 2 METHODS

We reported this pilot study based on the Checklist for Reporting Results of Internet E‐Surveys (CHERRIES) (24). The protocol was registered in ClinicalTrials.gov (NCT04391881) and approved by the Research Ethics Committee, Faculty of Dentistry, Cairo University, Egypt. After reviewing the literature about the pathogenesis of COVID‐19 and its related manifestations, we developed an online Arabic survey using Google form to target adults (over 18 years old) who live in Cairo, Egypt, via Facebook advertisement (Table S1).

Since it was not feasible to examine the patients clinically, and to avoid selection biases due to convenience sampling, all participants were volunteers to minimize the risk of bias. We included only adults diagnosed with COVID‐19 positive, confirmed with reverse transcriptase PCR (RT‐PCR), and are isolated in various hospitals in Cairo, Egypt, from May 15 to June 10, 2020.

We used a Google Form to create an online questionnaire containing multiple manifestations that were to be associated with COVID‐19 based on the available evidence. All symptoms included in this questionnaire were in four primary categories; the first category was the gustatory disorders described as a loss of salt sensation, loss of sweetness, and altered food taste. The second category was the symptoms associated with salivary glands infection described as dry mouth (i.e., hyposalivation), difficulty swallowing, pain or swelling in the submandibular gland area, and pain or swelling in the parotid gland area. The third category was the oral mucosal changes (i.e., oral lesions) described as oral ulcers, spots of mouth or lips, tongue redness, gingival bleeding, and burning sensation. The last category was the patients with no symptoms related to the oral cavity and salivary glands.

### 2.1 Statistical methods

We presented the categorical data as frequencies and percentages and analyzed using Fisher's exact test. The significance level was set at *p* ≤ 0.05 within all tests, and *p*‐values were corrected for multiple comparisons using Bonferroni correction. Statistical analysis were conducted with Windows version 4.0.3 of the R software (Team, 2020).

## 3 RESULTS

A total of 58 (53.4% males and 46.6% females) COVID‐19 patients ages 18–46 years were included in this pilot study, while 13 (22.4%) were healthcare workers, and there were no significant differences in age or gender between participants (*p* = 0.094 and 0.580 respectively; Table 1).

**TABLE 1:** Demographic data

Parameter		Frequency (n)	Percentage (%)	*p*‐Value
Gender	Male	31	53.4%	0.580
	Female	27	46.6%	
Age	≤30 years	34	58.6%	0.094
	>30 years	24	41.4%	
Job	Health worker	13	22.4%	<0.001*
	Non‐health worker	31*	53.4%	
	Not working	14	24.1%	

* Statistically significant (*p* ≤ 0.05).

**FIGURE 1:**
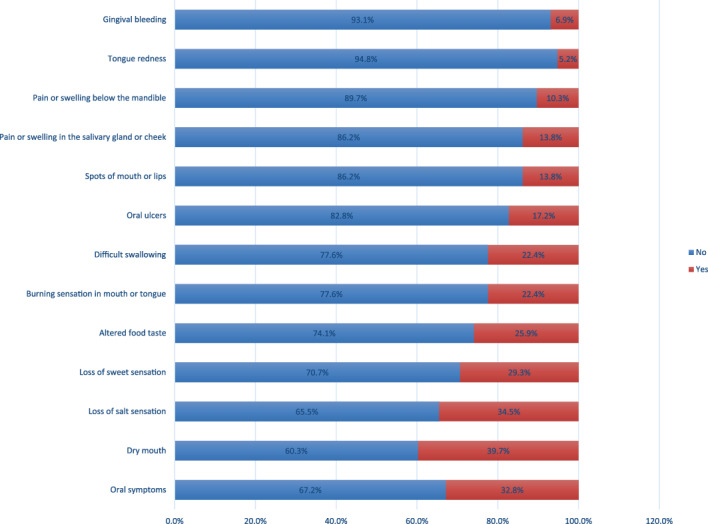
Prevalence of oral manifestations associated with COVID‐19 patients

Our results found that 67.2% of the patients had at least one of the oral manifestations, while about 32.8% (*n* = 19) did not have any symptoms related to the oral cavity. The high prevalence symptom was dry mouth 39.7% (*n* = 23), followed by gustatory dysfunction as 34.5% (*n* = 20) loss of salt sensation, 29.3% (*n* = 17) loss of sweet sensation, and 25.9% (*n* = 15) altered food taste. The prevalence of symptoms associated with salivary glands infection was 22.4% (*n* = 13) difficult swelling, 13.8% (*n* = 8) pain or swellings in the salivary gland or cheek, and 10.3% (*n* = 6) pain or swelling below mandible. While the oral mucosal changes were less prevalent as 22.4% (*n* = 13) of burning mouth sensation, 17.2% (*n* = 10) of ulceration, 13.8% (*n* = 8) of mouth and lip spots, 8.8% (*n* = 5) of tongue redness, and 7% (*n* = 4) of gingival bleeding (Figure 1).

**FIGURE 2:**
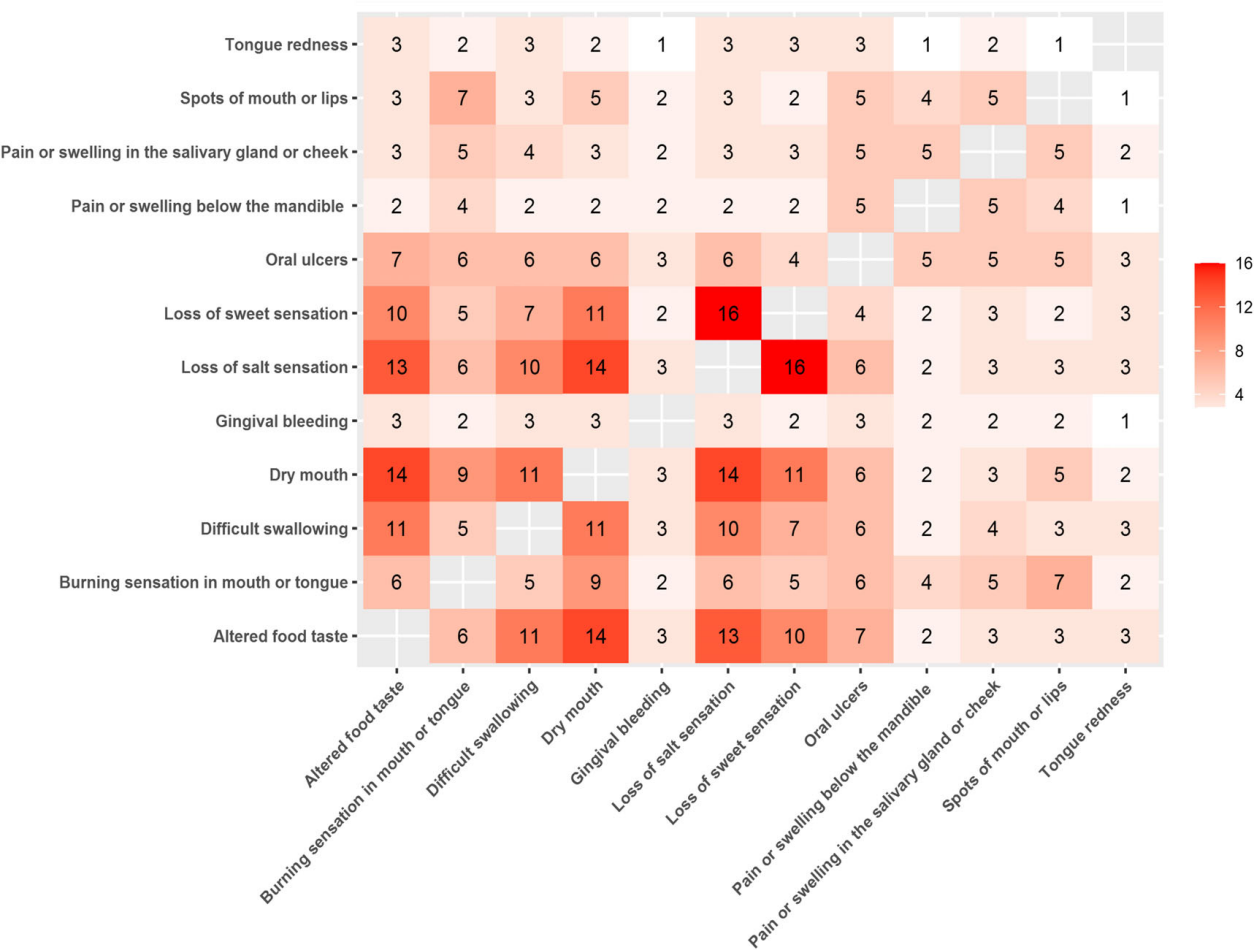
Heat‐map showing the oral symptoms correlated with each other

The most associated symptoms were loss of salt and sweetness, as observed in 16 (27.6%) participants, while the dry mouth and gustatory impairment symptoms were often associated in 14 (27.6%) participants for each manifestation. However, the association between the tongue's redness either with lips or mouth spots, discomfort or swelling below the mandible, or gingival bleeding, was the least frequent, which occurred in one case (Figure 2).

However, there was no significant association between the incidence of oral symptoms and demographic data (age, gender, or job) of the patients (*p* > 0.05; Table 2).

**TABLE 2:** Association between the occurrence of oral symptoms and status of different demographic aspects

Symptoms	Parameter		No		Yes		*p*‐Value
			*n*	%	*n*	%	
Oral symptoms	Gender	Male	21	67.7%	10	32.3%	0.431
		Female	18	66.7%	9	33.3%	
	Age	≤30 years	19	55.9%	15	44.1%	0.474
		>30 years	20	83.3%	4	16.7%	
	Job	Health worker	7	53.8%	6	46.2%	0.175
		Non‐health worker	22	71.0%	9	29.0%	
		Not working	10	71.4%	4	28.6%	
Dry mouth	Gender	Male	18	58.1%	13	41.9%	0.870
		Female	17	63.0%	10	37.0%	
	Age	≤30 years	23	67.6%	11	32.4%	0.492
		>30 years	12	50.0%	12	50.0%	
	Job	Health worker	8	61.5%	5	38.5%	0.785
		Non‐health worker	17	54.8%	14	45.2%	
		Not working	10	71.4%	4	28.6%	
Loss of salt sensation	Gender	Male	21	67.7%	10	32.3%	0.798
		Female	17	63.0%	10	37.0%	
	Age	≤30 years	24	70.6%	10	29.4%	0.988
		>30 years	14	58.3%	10	41.7%	
	Job	Health worker	8	61.5%	5	38.5%	0.358
		Non‐health worker	20	64.5%	11	35.5%	
		Not working	10	71.4%	4	28.6%	
Loss of sweet sensation	Gender	Male	21	67.7%	10	32.3%	1
		Female	20	74.1%	7	25.9%	
	Age	≤30 years	25	73.5%	9	26.5%	0.739
		>30 years	16	66.7%	8	33.3%	
	Job	Health worker	8	61.5%	5	38.5%	0.171
		Non‐health worker	23	74.2%	8	25.8%	
		Not working	10	71.4%	4	28.6%	
Altered food taste	Gender	Male	24	77.4%	7	22.6%	0.715
		Female	19	70.4%	8	29.6%	
	Age	≤30 years	27	79.4%	7	20.6%	0.181
		>30 years	16	66.7%	8	33.3%	
	Job	Health worker	10	76.9%	3	23.1%	0.295
		Non‐health worker	21	67.7%	10	32.3%	
		Not working	12	85.7%	2	14.3%	
Burning sensation in mouth or tongue	Gender	Male	21	67.7%	10	32.3%	0.431
		Female	24	88.9%	3	11.1%	
	Age	≤30 years	28	82.4%	6	17.6%	0.497
		>30 years	17	70.8%	7	29.2%	
	Job	Health worker	12	92.3%	1	7.7%	0.669
		Non‐health worker	22	71.0%	9	29.0%	
		Not working	11	78.6%	3	21.4%	
Difficult swallowing	Gender	Male	26	83.9%	5	16.1%	0.918
		Female	19	70.4%	8	29.6%	
	Age	≤30 years	29	85.3%	5	14.7%	0.547
		>30 years	16	66.7%	8	33.3%	
	Job	Health worker	11	84.6%	2	15.4%	0.756
		Non‐health worker	22	71.0%	9	29.0%	
		Not working	12	85.7%	2	14.3%	
Oral ulcers	Gender	Male	25	80.6%	6	19.4%	0.361
		Female	23	85.2%	4	14.8%	
	Age	≤30 years	29	85.3%	5	14.7%	0.911
		>30 years	19	79.2%	5	20.8%	
	Job	Health worker	12	92.3%	1	7.7%	0.508
		Non‐health worker	24	77.4%	7	22.6%	
		Not working	12	85.7%	2	14.3%	
Spots of mouth or lips	Gender	Male	26	83.9%	5	16.1%	0.811
		Female	24	88.9%	3	11.1%	
	Age	≤30 years	30	88.2%	4	11.8%	1
		>30 years	20	83.3%	4	16.7%	
	Job	Health worker	11	84.6%	2	15.4%	0.914
		Non‐health worker	27	87.1%	4	12.9%	
		Not working	12	85.7%	2	14.3%	
Pain or swelling in the salivary gland or cheek	Gender	Male	27	87.1%	4	12.9%	1
		Female	23	85.2%	4	14.8%	
	Age	≤30 years	31	91.2%	3	8.8%	0.864
		>30 years	19	79.2%	5	20.8%	
	Job	Health worker	12	92.3%	1	7.7%	0.902
		Non‐health worker	27	87.1%	4	12.9%	
		Not working	11	78.6%	3	21.4%	
Pain or swelling below the mandible	Gender	Male	28	90.3%	3	9.7%	0.300
		Female	24	88.9%	3	11.1%	
	Age	≤30 years	31	91.2%	3	8.8%	0.431
		>30 years	21	87.5%	3	12.5%	
	Job	Health worker	12	92.3%	1	7.7%	0.571
		Non‐health worker	28	90.3%	3	9.7%	
		Not working	12	85.7%	2	14.3%	
Tongue redness	Gender	Male	30	96.8%	1	3.2%	0.852
		Female	25	92.6%	2	7.4%	
	Age	≤30 years	32	94.1%	2	5.9%	0.700
		>30 years	23	95.8%	1	4.2%	
	Job	Health worker	12	92.3%	1	7.7%	0.505
		Non‐health worker	30	96.8%	1	3.2%	
		Not working	13	92.9%	1	7.1%	
Gingival bleeding	Gender	Male	30	96.8%	1	3.2%	0.840
		Female	24	88.9%	3	11.1%	
	Age	≤30 years	31	91.2%	3	8.8%	0.573
		>30 years	23	95.8%	1	4.2%	
	Job	Health worker	12	92.3%	1	7.7%	0.975
		Non‐health worker	30	96.8%	1	3.2%	
		Not working	12	85.7%	2	14.3%	

## 4 DISCUSSION

COVID‐19 patients have a wide variety of signs and symptoms, so the study of these manifestations will contribute to the early diagnosis and isolation of infected patients (Vaira et al., 2020). Also, COVID‐19 as an acute infection with multiple therapeutic measures could adversely affect oral health leading to opportunistic infections (e.g., recurrent herpes simplex virus (HSV‐1) infection and oral ulcerations) due to the compromised immune system and xerostomia associated with the reduced salivary flow (Dziedzic & Wojtyczka, 2020).

This pilot study revealed a high prevalence of salivary gland‐related symptoms supporting the beneficial effect of saliva on virus detection and the negative impact of saliva on viral transmission hypotheses (Han & Ivanovski, 2020; Xu et al., 2020). The effect of COVID‐19 on salivary glands was reflected in different ways: first patients complained of pain or swelling in either parotid or submandibular areas (13.8% and 10.3%, respectively), and second 39.7% suffered from a dry mouth. These findings indicates the protective role of saliva which continuously cleans the oral cavity and antiviral response against COVID‐19 (Farshidfar & Hamedani, 2020; Malamud et al., 2011).

Hyposalivation might be a potential complication of chronic sialadenitis caused by COVID‐19. As a repair mechanism following the inflammatory destruction of the salivary glands, this destruction could be repaired by fibrosis affecting both the acini and the duct leading to hypo‐secretion. Hyposalivation raises the risk of inorganic salts deposition on the ductal wall that induces sialolithiasis contributing to stenosis and dilatation of the ducts (Wang et al., 2020). Respiratory infection incidence will then increase by enhancing virus adhesion and colonization and destroying the oral mucosa surfaces and airways, thus, decreasing antimicrobial peptides and proteins (Han & Ivanovski, 2020; Iwabuchi et al., 2012).

However, T‐cell receptors for human pathogenic saliva can be the initial portal of the virus to the body; thus, the proliferation of the COVID‐19 in the salivary gland may be responsible for the spread of infection from asymptomatic patients (Liu et al., 2011; Zhang et al., 2020). As well, salivary glands can act as a harbor for a latent infection that can be reactivated later with a risk of chronic sialadenitis (Wang et al., 2020); therefore, physicians should pay more attention to any variation in salivary glands or salivary secretions (Vinayachandran & Saravanakarthikeyan, 2020).

Taste loss (ageusia) occurs due to the viral infection to the olfactory cranial nerve or from rhinitis and nasal obstruction. The loss of smell (anosmia) is related to retro‐nasal olfaction which is a sensory process, combining ortho‐nasal smell and taste patterns and enabling people to perceive flavor. This mechanism is temporarily disrupted during upper respiratory infections due to mucosal inflammation and obstruction of the nasal passages, thereby physically blocking flavor and odor molecules from entering the olfactory cleft (Melley et al., 2020). In this study, consistent with the current evidence, olfactory dysfunction was the primary factor identified either with or without gustatory dysfunction (Amorim dos Santos et al., 2020; Passarelli et al., 2020).

Burning sensation of the mouth (22.4%) could be due to several reasons (e.g., candidal infection, dry mouth, oral ulceration, or drug‐induced). Evidence show that viral infection could weaken the immune system thus, creating secondary infections such as oral candidiasis. Candidiasis is the most prevalent opportunistic infection in HIV (Nokta, 2008) and is also reported in COVID‐19 (dos Santos et al., 2020; Vinayachandran & Saravanakarthikeyan, 2020).

Concerning the oral ulceration (17.2%), mouth and lip spots (13.8%), their prevalence was higher than other oral changes which supports the case report of ulceration and skin rash in COVID‐19 patients (Soares et al., 2020). As such, many patients might have the same symptoms undetected due to the lack of oral examination by healthcare professionals (Martín Carreras‐Presas et al., 2020). Another case report of oral ulceration revealed that the history of the lesion was a 24‐hr painful inflammation of the tongue papilla, followed by 24‐hr erythematous macula, which evolved into irregular and asymptomatic ulcers; hence, many patients might not be aware of having oral ulceration due to lack of pain (Chaux‐Bodard et al., 2020). Moreover, a careful oral examination is recommended to obtain accurate data about oral manifestations associated with COVID‐19. Ulcers and vesiculobullous lesions may be discussed from a different perspective, as case reports that found significant hemorrhage, vascular congestion, lymphocytic infiltration in the connective tissue, and different‐sized thrombi after the incisional biopsy (Cruz Tapia et al., 2020; Magro et al., 2020). Thus, the immune response to COVID‐19 stimulates the Langerhans' cells and lymphocytes, inducing vasculitis (lymphocytic thrombophilic arteritis; Guerini‐Rocco et al., 2020).

Although gingivitis and gingival bleeding are a common feature in HSV‐1 and In EVD (Ebola virus disease; Scully & Samaranayake, 2016), our results showed that they were less prevalent (6.9%) in symptoms associated with COVID‐19. However, the prevalence of tongue redness was 5.2%, indicating that viral infections may change in the coating and color of the tongue. The case report identified that a geographic tongue was associated with candidiasis and ulceration in COVID‐19 patients, which suggests that the geographic tongue in this pilot study was not specific to COVID‐19 (dos Santos et al., 2020).

On the other hand, this pilot study revealed that 32.8% of the patients had no symptoms related to the oral cavity or salivary glands, consistent with the suggested estimation of asymptomatic patients with COVID‐19 (Mizumoto et al., 2020; Nishiura et al., 2020).

Our study has further limitations. First, the main methodological limitation is the limited sample size since this is a pilot study. Second, this is an online questionnaire; thus, we have not been able to document the patient's full history and a clinical oral examination (e.g., length of infection, the severity of the case, oral hygiene, habits, and the taken medications), which might have a misleading impact on the results. Despite the limitations, this pilot study's findings could help understand the oral manifestations associated with COVID‐19.

## 5 CONCLUSION

Our study indicates a significant impact of COVID‐19 on the oral cavity based on limited sample size. Salivary gland‐related symptoms and taste disorders are high prevalence in COVID‐19 patients, while the detailed oral manifestations associated with COVID‐19 and their pathophysiology required further investigation. Further clinical studies with larger sample sizes and detailed patient's history, are required to confirm our results and clarify the full impact of COVID‐19 on the oral cavity and salivary glands.

## CONFLICT OF INTEREST

All authors declare no conflicts of interest.

## AUTHOR CONTRIBUTIONS

Conceptualization: D.M.E.; Methodology: D.M.E., E.A.G., and A.G.A.K.; Data collection: W.S.A., R.H.A., and R.A.H.; Data analysis: A.G.A.K., W.S.A.; Writing original draft preparation: A.G.A.K., E.A.G., D.M.E., R.H.A., and R.A.H.; Writing–review and editing: A.G.A.K., D.M.E., and E.A.G.; All authors have read and agreed to the published version of the manuscript.

## Supporting information


**Supplementary Table S1** An example of the online survey that we used

**Appendix** S1: Supporting Information

## Data Availability

The data that support the findings of this study are available on request from the corresponding author. The data are not publicly available due to privacy or ethical restrictions.
