# Supplementary material for: Oral manifestations of COVID‐19 patients: An online survey of the Egyptian population
Source: Clin Exp Dent Res. 2021 May 1;7(5):852–60. doi: 10.1002/cre2.429 (PMC8239795; doi:10.1002/cre2.429)
Supplement: Supplementary file 1 — Supplementary Table S1 An example of the online survey that we used [file CRE2-7-852-s002.docx]

Supplementary Table: An example of the online survey that we used

| 1. **Do you agree to participate in this questionnaire?** | 1. Yes 2. No |
| --- | --- |
| 1. **Name** | Free text |
| 1. **Phone** | Free text |
| 1. **Job** | 1. Healthcare workers 2. Non-healthcare workers |
| 1. **Nationality** | 1. Egyptian 2. Other |
| 1. **How was your diagnosis of COVID19 confirmed?**   **) you can select more than one answer)** | 1. Swab (PCR) 2. Lab investigations 3. Computed tomography |
| 1. **Did you have any of these symptoms during your illness?**   **) you can select more than one answer)** | 1. Bleeding from the gums 2. Spots or rash on the lips or inside the mouth 3. Pain or swelling in the salivary glands (in front of the ear) 4. Losing a sense of bitterness 5. Losing a sense of sweetness 6. Mouth ulcers 7. Burning tongue or mouth 8. Taste alteration 9. Tongue redness 10. Dry mouth 11. Difficulty in swallowing 12. Swelling below the lower jaw 13. Nothing |
